# Breast Cancer Evaluation and Targeted Investigational Therapy (BEAT-IT): A Pilot Prospective Tissue-Testing Study for Breast Cancer Clinical Trial Enrollment

**DOI:** 10.21203/rs.3.rs-10936036/v1

**Published:** 2026-10-14

**Authors:** Diego F. Chamorro, Evthokia Hobbs, Meghan Karuturi, Elyse Lopez, Lumarie Santiago, Rony Avritscher, Aysegul Sahin, Funda Meric-Bernstam, Bora Lim

## Abstract

Background Metastatic breast cancer (mBC) is heterogeneous not only clinically but genomically. Beyond 2–3 lines of therapy, many patients lack access to rationally selected investigational therapies. We evaluated whether a prospective, multi-omics assay from obtained tissue could 1) identify actionable alterations with rapid turnaround and 2) channel patients to biomarker-matched clinical trials, while maintaining procedural safety. Methods BEAT-IT was a prospective study in which core biopsies from patients with mBC (primary and metastatic lesions) were evaluated using immunohistochemistry (IHC), fluorescence in situ hybridization (FISH), and next-generation sequencing (NGS). Primary endpoint was the proportion of patients bearing actionable alterations and the proportion referred to biomarker-matched trials. Secondary endpoints included turnaround time (TAT) and biopsy-related morbidity. Proportions are reported with Wilson 95% confidence intervals (CIs). Exploratory subgroup comparisons including molecular subtype comparison were planned with chi-square or Fisher's exact tests. Results Among the 157 enrolled participants, the median TAT from biopsy to finalized report was 20 days. Actionable alterations were identified in 49.0% (77/157; 95% CI 41.3–56.8%). Overall, 49 of 157 patients (31.2%; 95% CI 24.5–38.8%) enrolled in at least one therapeutic clinical trial after profiling. A single grade 1 pneumothorax (1/162 biopsied patients, 0.6%) was the only biopsy-related complication. Retrospective annotation using current ESCAT criteria identified a tier I alteration in 20% of patients with tumor-informative DNA results. Conclusions Prospective, biopsy-triggered multi-omics profiling in mBC is feasible, safe, and delivers clinically actionable results within approximately 3 weeks. Our approach identified actionable alterations in approximately half of patients and supported enrollment in therapeutic clinical trials for nearly one-third. Trial registration: This study evaluated a diagnostic/implementation strategy without assignment to a therapeutic intervention; clinical-trial registration was not required.

### Background

Hanahan and Weinberg and many following discoveries in cancer noted complex, heterogeneous biology beyond the original six hallmarks of cancer. Each tumor expresses a distinct constellation of these hallmarks, underscoring the profound inter- and intra-tumoral heterogeneity that challenges the development of universal targeted therapies (1, 2). Precision oncology stems from the goal that characterizing these individualized molecular features, particularly through genomic and transcriptomic analyses, can guide the selection of more effective, personalized treatments (3).

Large-scale precision-medicine efforts have demonstrated the feasibility and clinical relevance of molecularly guided therapy. In the United States, the NCI-MATCH trial continues to assign patients to treatment arms based on actionable genomic alterations (4). In Europe, SAFIR02 demonstrated that prospective genomic profiling can direct approximately 28% of patients to genotype-matched trials (5). The success of tumor-agnostic agents, such as the TRK inhibitor larotrectinib, further validates the principle that targeting specific alterations, rather than tumor histology, can result in meaningful clinical benefit (6, 7).

However, genomics alone does not fully capture the functional drivers of metastatic breast cancer. Protein-level alterations, such as phosphatase and tensin homolog (PTEN) loss, c-mesenchymal-epithelial transition factor (c-MET) activation, and androgen receptor (AR) expression, may not be detectable through DNA sequencing but remain actionable vulnerabilities (8–10). Thus, integrating proteomic and genomic assays may reveal a broader spectrum of therapeutically relevant abnormalities. Furthermore, prospective biopsy at the time of metastatic progression allows assessment of real-time tumor biology, which may differ substantially from archival primary-tumor specimens commonly used in retrospective analyses.

We hypothesized that a biopsy-triggered, multi-omic workflow incorporating both proteomic and genomic assays would improve identification of actionable targets and increase allocation to biomarker-matched clinical trials for patients with mBC. To test this hypothesis, we implemented a prospective tissue-acquisition protocol using CLIA-validated assays with the goal of directing patients to genotype- and phenotype-informed therapeutic trial options or standard-of-care regimens. Our study also evaluated the feasibility, safety, and real-world clinical utility of this precision-oncology pipeline.

### Methods

## Study Objectives

We conducted a prospective, single-center implementation study at The University of Texas MD Anderson Cancer Center (MDACC). The primary aim was to determine whether multi-omic profiling of prospectively collected mBC biopsies using CLIA-approved assays could identify actionable alterations and guide allocation to biomarker-matched clinical trials. Secondary aims included characterizing genomic and proteomic alterations in metastatic lesions and assessing feasibility, tissue adequacy, and procedural safety.

### Participants

Eligible participants were adults (female and male) with stage IV breast cancer, either de novo or recurrent, with at least one metastatic or primary lesion safely amenable to image-guided biopsy. Patients were referred by their treating oncologist and enrolled with informed consent. All participants agreed to undergo a mandatory research biopsy and to be potentially assigned to genotype- or phenotype-matched therapeutic trials or standard-of-care treatment based on assay results. Clinical metadata collected included age, receptor subtype (ER/PR/HER2), biopsy site, and survival outcomes. Of 200 referred and screened patients, 162 underwent research biopsy, and 157 patients with confirmed mBC and analyzable tissue constituted the analytic cohort. The enrollment period was January 2012 to October 2013, with survival follow-up through April 14, 2021.

### Biopsy Procedure and Tissue Acquisition

All biopsies were performed by interventional radiology. In a single procedure, four core-needle biopsy (CNB) and four fine-needle aspiration (FNA) samples were obtained from the safest and most accessible metastatic lesion. If CNB could not be obtained, eight FNAs were collected. Samples were immediately processed per MDACC standard operating protocols for IHC, FISH, and NGS.

### Assay Validation and Platform Expansion

All assays in this study were performed in CLIA-certified laboratories at MD Anderson Cancer Center. NGS variant calls had undergone prior orthogonal validation during assay development and were clinically reported. Biopsy-handling protocols were optimized for challenging metastatic sites, such as bone and liver, where stromal admixture and decalcification may impair nucleic acid integrity. The targeted alteration detection of PTEN, AR, c-MET, ALK were performed internally.

### Immunohistochemistry and Cytogenetic Assays

IHC was performed to assess expression of PTEN, AR, c-MET, and anaplastic lymphoma kinase (ALK). Both ALK and c-MET, as selective predictive biomarkers, were evaluated by FISH for amplification, deletion, or loss. Three markers, PTEN, AR, and c-MET, were included because of their potential relevance for targeted therapies, although they are not part of routine breast cancer clinical testing.

## Next-Generation Sequencing

A targeted PCR-based NGS platform was used to screen for frequently reported point mutations in the genes listed in Supplemental Table 2. This analysis contained either 11 genes (CMS11) or 46 genes (CMS46) based on available platforms. Assays were conducted in the CLIA-certified Molecular Diagnostic Laboratory (MDL) at MDACC (11). The analytical sensitivity of the CMS11 and CMS46 assays for single-nucleotide variants (SNVs) was determined to be 5 to 10%, corresponding to the detection of one mutant allele in a background of 19 wild-type alleles (5%) to one mutant allele in nine wild-type alleles (10%). These thresholds were established based on sequencing depth at each base and the ability to confirm low-frequency variants using independent orthogonal platforms. All NGS findings were further validated during assay development in our CLIA-certified MDL; however, mutations outside the targeted regions could not be detected using these panels. Matched non-tumor tissue was not analyzed; therefore, a germline origin for detected variants could not be excluded. Silent mutations (those not resulting in an amino acid change) were not reported. Hotspot mutation profiling was additionally performed on formalin-fixed, paraffin-embedded metastatic breast cancer tissue using a 46- or 50-gene NGS panel, or an 11-gene Sequenom panel, as previously described (11, 12). Detected alterations were retrospectively annotated according to the ESMO Scale for Clinical Actionability of molecular Targets (ESCAT), applying current tier assignments for advanced breast cancer (29, 30); annotation reflects present-day evidence rather than evidence available at enrollment.

## Statistical Analysis

Continuous variables were summarized with means, standard deviations, medians, and ranges; categorical variables with counts and proportions. The survival endpoint was overall survival (OS), defined as time from biopsy (recurrent disease) or diagnosis (de novo stage IV) to death from any cause; patients without events were censored at last follow-up. Analysis populations were defined as follows: the analytic cohort comprised all 157 patients with confirmed mBC; assay results were reported against per-assay evaluable denominators; and DNA-profiling results were reported at the test level for all targeted DNA tests attempted. A DNA test was considered tumor-informative if there was actionable alteration (at the time of active study). OS was estimated using Kaplan-Meier methodology, and multivariable associations were evaluated using Cox proportional-hazards regression. All statistical tests were two-sided with alpha = 0.05. Given the exploratory nature of this implementation study, no formal power calculation was performed.

### Ethics and Consent

The study protocol (LAB11-0337) was approved by the MDACC Institutional Review Board (IRB). All participants provided written informed consent for biopsy, molecular testing, and the use of clinical and genomic data for research.

### Results

## Patient Characteristics and Biopsy Feasibility

Between January 2012 and October 2013, 200 patients were screened and 162 provided informed consent for prospective research biopsy (Fig. 1). Thirty-eight patients did not undergo biopsy (biopsy infeasible per radiologist or treating physician, n = 12; insurance denial, n = 6; cancellation, n = 5; consent withdrawal, n = 3; staging workup negative for metastatic disease, n = 2; protocol ineligibility, n = 1; other reasons, n = 9). Five patients were excluded following biopsy due to non-breast malignancy (one patient was diagnosed with non-small cell lung cancer) or insufficient sample (n = 4), leaving 157 patients with confirmed mBC in the analytic cohort. Baseline clinical characteristics of referred patients are detailed in Table 1.

Table 1
Clinical Characteristics

| Characteristic | N (%) |
| --- | --- |
| **Age at enrollment (n = 157)** | |
| Median | 52 |
| Range | 27–81 |
| **Race (n = 157)** | |
| Asian | 7 (4.5%) |
| Black | 19 (12.1%) |
| Other | 2 (1.3%) |
| Spanish/Hispanic | 20 (12.7%) |
| White | 109 (69.4%) |
| **Sex (n = 157)** | |
| Female | 155 (98.7%) |
| Male | 2 (1.3%) |
| **Menopausal status at diagnosis (n = 155)** | |
| Peri | 2 (1.5%) |
| Post | 63 (40.1%) |
| Pre | 90 (57.3%) |
| **Molecular subtype at recurrence (n = 157)** | |
| HR-/HER2+ | 6 (3.8%) |
| HR+ | 96 (61.1%) |
| HR+/HER2+ | 6 (3.8%) |
| TNBC | 49 (31.2%) |
| **De novo metastatic disease (n = 157)** | |
| No | 124 (79.0%) |
| Yes | 33 (21.0%) |
| **Survival status since consent (n = 157)** | |
| Alive | 25 (15.9%) |
| Deceased | 132 (84.1%) |

Biopsies were obtained from a range of metastatic and locoregional sites, most commonly the liver (56/157, 36%), lymph nodes (32/157, 20%), bone (22/157, 14%), and lung (11/157, 7%) (Table 2). Adequate tissue for at least one molecular assay was obtained in 154 of 157 patients (98%).

Table 2
Sample adequacy by organ sites and assessed biomarkers.

| Characteristic | N (%) |
|---|---|
| **Patients enrolled (n = 200)** | |
| **Did not complete biopsy** | 42 (21.0%) |
| Research tissue not collectable per radiologist | 12 (6.0%) |
| Insurance approval denied | 6 (3.0%) |
| Patient cancelled biopsy | 5 (2.5%) |
| Insufficient tumor per pathologist | 4 (2.0%) |
| Admitted to emergency center | 3 (1.5%) |
| Withdrew consent | 3 (1.5%) |
| Returned home, lost to follow-up | 2 (1.0%) |
| Biopsy not recommended by treating physician | 2 (1.0%) |
| No malignant cells on preliminary pathology | 1 (0.5%) |
| Biopsy site changed to pulmonary service | 1 (0.5%) |
| Staging studies negative for MBC | 1 (0.5%) |
| Biopsy missed due to study staff coverage | 1 (0.5%) |
| Protocol violation (patient consented with mild cognitive disorder) | 1 (0.5%) |
| **Completed biopsy** | 158 (79.0%) |
| Negative for metastatic breast cancer | 1 (0.6%) |
| Insufficient tumor for IHC | 22 (13.9%) |
| Complications caused by biopsy | **[n (%)]** |
| **Biopsy site (n = 157)** | |
| Liver | 56 (35.7%) |
| Lymph node | 34 (21.7%) |
| Bone | 22 (14.0%) |
| Skin/soft tissue | 21 (13.4%) |
| Lung | 11 (7.0%) |
| Breast | 4 (2.5%) |
| Other | 9 (5.7%) |

| Characteristic | N (%) |
|---|---|
| **Patients enrolled (n = 200)** | |
| **PTEN IHC (n = 157)** | |
| Positive | 110 (70.1%) |
| Negative | 19 (12.1%) |
| Not evaluable | 28 (17.8%) |
| **AR IHC (n = 157)** | |
| Positive | 89 (56.7%) |
| Negative | 45 (28.7%) |
| Not evaluable | 23 (14.6%) |

## Proteomic and Cytogenetic Profile

Evaluable results varied by marker. PTEN expression was intact in 110 of 129 evaluable patients (85%) and lost in 19 (15%); 28 samples were not evaluable. AR expression was detected in 89 of 134 evaluable patients (66%) and absent in 45 (34%); 23 samples were not evaluable. c-MET expression was positive in 20 of 131 evaluable patients (15%), negative in 108, and equivocal in 3; 26 samples were not evaluable. FISH identified c-MET amplification in 1 of 114 evaluable patients and ALK positivity in 2 of 129 evaluable patients (Table 2).

## Next-Generation Sequencing and Mutation Landscape

Targeted DNA profiling was attempted in 111 tests (81 by NGS panel, 30 by Sequenom). A tumor-informative result was obtained in 59 tests (53%); the remaining 52 were noninformative, most commonly because of insufficient tumor DNA. Among patients with informative results, 38 harbored at least one pathogenic mutation and 23 had at least one variant of uncertain significance; these categories were not mutually exclusive. Across all platforms (CMS11, CMS46, and Sequenom), 45 mutations were identified in 38 patients (Table 3).

Table 3

Patients who underwent the NGS and various subgroup.

Molecular profiling demonstrated marked subtype-specific heterogeneity (Fig. 2A). Hormone receptor (HR)-positive tumors showed enriched AR expression, while PTEN loss and MET-pathway abnormalities were more frequent in triple-negative breast cancer (TNBC) and HER2-positive disease. TP53 and

PIK3CA were the most mutated genes, substantially more prevalent than other alterations (Fig. 2B and 2C; Supplemental Fig. 1). Five patients demonstrated co-mutations in TP53 and PIK3CA.

Two germline polymorphisms were detected, a MET codon 375 variant (TNBC) and an APC codon 870 variant (HR+/HER2-), shown in Fig. 2D. An OncoPrint of 23 cancer-associated genes (Supplemental Table 2) illustrated the wide inter-patient molecular diversity characteristic of mBC, even among different mBC subtypes (Supplemental Fig. 1).

Retrospective annotation of detected alterations by current ESCAT criteria demonstrated durable clinical relevance of the profiling output (Table 4). Twelve patients (7.6% of the cohort; 20.3% of the 59 with tumor-informative DNA results) harbored a tier I alteration, all PIK3CA mutations in HR-positive/HER2-negative disease, and 22 patients (14.0%; 37.3% of informative) harbored at least one tier I to III genomic alteration. PTEN loss by IHC, identified in 19 patients including 7 with HR-positive/HER2-negative disease, represents a protein-level counterpart of the PIK3CA/AKT1/PTEN axis now targetable with capivasertib. Because the era's panels did not include ESR1 or BRCA1/2, both currently tier I in this setting, these figures underestimate the tier I prevalence that contemporary panels would identify.

Table 4
Retrospective ESCAT annotation of molecular alterations detected in the study cohort, applying current ESMO tier assignments for advanced breast cancer (29, 30).

| **Alteration (assay)** | **Patients, n** | **Subtype context** | **ESCAT tier** | **Contemporary therapeutic implication** |
|---|---|---|---|---|
| PIK3CA mutation (NGS) | 15<br>(12 HR+/HER2-,<br>3 TNBC) | HR+/HER2- | I-A | Alpelisib-fulvestrant (SOLAR-1); inavolisib-based triplet; capivasertib-fulvestrant |
| PTEN alteration (NGS mutation; IHC loss as protein-level counterpart) | Mutation 1 (HR+/HER2-); IHC loss 19 of 129 evaluable (7 HR+/HER2-, 11 TNBC, 1 HER2+) | HR+/HER2- | I/II | Capivasertib-fulvestrant (CAPItello-291); IHC loss annotated as protein-level proxy, trial biomarker was genomic |
| AKT1 mutation (NGS) | 0 detected | HR+/HER2- | I/II | Capivasertib-fulvestrant; hotspot covered by panel, none detected |
| ERBB2 activating mutation (NGS) | 1 (HR+/HER2-) | Any | II-B | Neratinib (SUMMIT) |
| KRAS mutation (NGS) | 2 (HR+/HER2-) | Any | III-A | Validated in other tumor types; investigational in breast |
| FGFR3 mutation (NGS) | 2 (HR+/HER2-) | Any | III-A | FGFR inhibition validated in urothelial carcinoma |
| RET mutation (NGS) | 1 (TNBC) | Any | III | Selective RET inhibition validated for RET-altered tumors |
| MET mutation (NGS) / MET amplification (FISH) | 1 / 1 of 114 evaluable (both HR+/HER2-) | Any | III | MET inhibition validated in NSCLC |
| ALK positivity (FISH) | 2 of 129 evaluable | Any | IV | Hypothetical target; fusion status not characterized |

Counts are patient-level. Annotation is retrospective; tier assignments reflect current evidence, not evidence available at enrollment (2012 to 2013). The two germline polymorphisms (MET codon 375 and APC codon 870) were excluded from actionability annotation. ESR1 and BRCA1/2 were not covered by the panels used; because both are now tier I in this setting, the tier I prevalence reported here is an underestimate of what contemporary panels would identify. Protein-level assays (PTEN, AR, and c-MET IHC; ALK and c-MET FISH) are not formally classifiable by ESCAT, which ranks genomic alterations, and are shown alongside their genomic counterparts.

| Alteration (assay) | Patients, n | Subtype context | ESCAT tier | Contemporary therapeutic implication |
|---|---|---|---|---|
| AR expression (IHC) | 89 of 134 evaluable (66%) | All subtypes | Not ESCAT-classifiable (protein biomarker) | AR-directed therapy investigational in breast cancer |
| TP53 and other non-actionable mutations (HRAS, SMAD4, NRAS, KIT, PDGFRA) | TP53 17; others 5 (1 each HRAS, SMAD4, NRAS, KIT, PDGFRA) | Any | X | No clinical actionability in breast cancer |

Counts are patient-level. Annotation is retrospective; tier assignments reflect current evidence, not evidence available at enrollment (2012 to 2013). The two germline polymorphisms (MET codon 375 and APC codon 870) were excluded from actionability annotation. ESR1 and BRCA1/2 were not covered by the panels used; because both are now tier I in this setting, the tier I prevalence reported here is an underestimate of what contemporary panels would identify. Protein-level assays (PTEN, AR, and c-MET IHC; ALK and c-MET FISH) are not formally classifiable by ESCAT, which ranks genomic alterations, and are shown alongside their genomic counterparts.

## Turnaround Time of Molecular Assays

The TAT was clinically actionable across all platforms. For IHC, the median TAT was 20 days (range 2-121); for FISH, the median TAT was 20 days (range 5–99); and for NGS, the median TAT was 21 days (range 8-101). Summarized TAT for each assay is shown in Supplemental Table 3.

## Allocation to Biomarker-Matched Clinical Trials

Following molecular profiling, 49 of 157 patients (31.2%; 95% CI 24.5–38.8%) enrolled in at least one therapeutic clinical trial, accounting for 70 therapeutic-trial participations across 40 unique protocols (Supplemental Table 1). An additional 30 patients participated in nontherapeutic laboratory or observational protocols, accounting for 52 nontherapeutic participations; in total, 60 unique patients (38.2%) accounted for 122 trial participations. Among the 40 therapeutic protocols, 20 incorporated targeted agents directed at alterations identified in this study.

The therapeutic-trial enrollment rate observed in this cohort (31.2%) is comparable or favorable to allocation rates reported by large precision-oncology programs such as MSK-IMPACT and SAFIR02 (16, 17), as summarized in Supplemental Table 4. These findings reinforce the clinical actionability of a prospective, multi-omic profiling strategy in mBC.

## Survival Outcomes

During a median follow-up of 14.7 months (range 0.1-100.8), 132 of 157 patients died; median OS was 17.5 months (95% CI 14.3–20.5) (Fig. 3A). Overall survival did not differ between patients who did and did not enroll in a clinical trial (log-rank p = 0.721).

Multivariable Cox modeling (concordance = 0.73, global p = 0.003) identified ER positivity as the dominant protective factor (HR 0.16, $p < 0.001$). PR positivity showed a nonsignificant trend toward increased risk (HR 2.52, p = 0.08). Trial enrollment was not associated with overall survival (HR 1.14, p = 0.70) after adjustment, suggesting preferential enrollment of patients with biologically aggressive disease rather than harm from the experimental therapy while further validation is needed (Supplemental Fig. 2).

Survival varied substantially by tumor subtype: HER2-positive patients (n = 12) showed a median OS (mOS) of 42.7 months; HR-positive patients (n = 96), 22.4 months; and patients with TNBC (n = 49), 8.2 months. Menopausal status, race, HER2 status, PR status, and clinical-trial enrollment were not independently associated with OS in multivariable analysis (Fig. 3B).

## Discussion

Metastatic breast cancer remains a major clinical challenge, with survival improvements lagging far behind advances achieved in early-stage disease. This gap reflects the marked biological heterogeneity of metastatic tumors and the frequent discrepancy between primary and metastatic lesions (13). Our study suggests that a biopsy-triggered, multi-omic profiling strategy performed in a CLIA-certified setting is both feasible and clinically informative, with a tissue-adequacy rate and turnaround time comparable to or exceeding those reported in large national precision-oncology programs (14, 15).

In our cohort, prospective biopsies yielded actionable biomarkers in nearly half of patients and supported therapeutic clinical trial enrollment in nearly one-third of cases. This enrollment rate is consistent with those reported by other precision-oncology efforts, such as MSK-IMPACT (11%) and SAFIR02 (16%) (16, 17). Importantly, incorporating additional proteomic assays, as many studies focus mainly on genomic assays, identified clinically relevant vulnerabilities not captured by genomic testing alone. These findings support the growing recognition that DNA-only approaches may underestimate the functional complexity of metastatic tumors and that integrated multi-omic profiling can broaden therapeutic opportunities (18, 19). Retrospective ESCAT annotation reinforces this point in both directions: alterations detected on the era's limited panels map onto contemporary tier I therapies (PIK3CA, and the PIK3CA/AKT1/PTEN axis via capivasertib), while the protein-level PTEN, AR, and MET assays anticipate the ongoing integration of non-genomic biomarkers into actionability frameworks. At the same time, the absence of ESR1 and BRCA1/2 coverage on those panels illustrates how rapidly the actionable target space has expanded, and by extension the value of profiling infrastructure that can be re-annotated as evidence evolves.

Procedure-related safety was excellent, with a single grade 1 pneumothorax (1/162, 0.6%) that resolved with overnight observation and no other procedure-related complications, aligning with established data on image-guided research biopsies (20, 21). The median 20-day turnaround time for IHC, FISH, and NGS results further underscores the feasibility of implementing this workflow within real-world clinical timelines. Together, these operational metrics highlight the feasibility of embedding prospective biopsies into routine care for mBC.

Our survival analysis reflects the expected prognostic landscape of metastatic disease, with HER2-positive patients achieving the most favorable outcomes and TNBC patients demonstrating the poorest survival (22, 23). Trial participation was not independently associated with OS after adjustment for biological covariates, suggesting that higher crude mortality in the trial-enrolled cohort primarily reflects selection of patients with more aggressive disease rather than harm from investigational therapies. In addition, given the era in which the trial was conducted, effective targeted therapy options were limited.

Although other strategies, such as liquid biopsy, are being incorporated into precision-oncology workflows using ctDNA profiling and minimally invasive sampling, they have a major limitation: they cannot match the biological resolution of tissue-based analysis (24, 25). Proteomic assays, including PTEN, AR, and MET analysis, as well as phospho-protein signaling measurements, require intact cellular architecture and cannot be captured through cell-free analytes (26–28). In addition, transcriptomic profiling, particularly fusion detection, pathway-level expression signatures, and tumor-microenvironment characterization, remains far more robust and reliable with tissue-based techniques than in plasma. Liquid biopsy also lacks spatial context and cannot distinguish tumor-cell-intrinsic alterations from stromal or immune-cell contributions (13). For these reasons, prospective tissue biopsy remains essential for comprehensive multi-omic profiling, enabling integrated genomic, proteomic, and transcriptomic analyses to identify a broader landscape of actionable targets in mBC - although continued innovation may bring liquid biopsy as the mainstay of multi-omic information.

Among the limitations of this study, it is relevant to note that it was a single-center study without a contemporaneous control arm and that our findings may not fully generalize to community settings, where biopsy logistics and assay availability may differ from academic practice. Second, matched normal tissue was not analyzed, precluding definitive classification of germline versus somatic variants. Third, although multi-omic profiling increased the detection of actionable alterations, not all identified targets corresponded to available clinical-trial options at the time of enrollment. Finally, while this study demonstrates feasibility and clinical utility of rapid biopsy and multi-omic assay resulting in precision allocation of treatment options, the study was not powered to detect survival differences. In addition, enrollment occurred in 2012 and 2013, when targeted hotspot panels of 11 to 50 genes represented standard practice; contemporary comprehensive genomic and transcriptomic platforms interrogate a far broader target space, and these findings should be interpreted as proof of concept for the biopsy-triggered multi-omic workflow rather than as a benchmark of current assay performance. Despite the aforementioned limitations, this pilot study provides meaningful real-world evidence supporting the integration of prospective metastatic biopsies and multi-omic profiling that enables true precision oncology workflows.

### Conclusions

This study validates the concept that prospective biopsy-triggered, multi-omic profiling of mBC is feasible, safe, and clinically actionable in a real-world clinical setting. Fresh biopsies from metastatic sites can provide sufficient tissue for molecular assays in nearly all patients, including assays that might require fresh tissue. Rapid turnaround times additionally support timely therapeutic decision-making. Integrated genomic and proteomic analysis broadened the spectrum of detectable actionable alterations and facilitated therapeutic clinical trial participation for nearly one-third of patients. These findings highlight the value of real-time analysis of metastatic tissue and the incorporation of multi-omic platforms into precision oncology in mBC patients. Continued evolution of multi-omic technologies and effective clinical trial options will be essential to further refine personalized care strategies and improve outcomes for patients with mBC based on this foundation.

### Abbreviations mBC
metastatic breast cancer
IHC
immunohistochemistry
FISH
fluorescence in situ hybridization
NGS
next-generation sequencing
TAT
turnaround time
PTEN
phosphatase and tensin homolog
c-MET
c-mesenchymal-epithelial transition factor
AR
androgen receptor
CLIA
Clinical Laboratory Improvement Amendments
MDACC
MD Anderson Cancer Center
CNB
core-needle biopsy
FNA
fine-needle aspiration
cfDNA
circulating cell-free DNA
ALK

anaplastic lymphoma kinase
MDL
Molecular Diagnostic Laboratory
PCR
polymerase chain reaction
OS
overall survival
mOS
median overall survival
PFS
progression-free survival
TCGA
The Cancer Genome Atlas
HR
hormone receptor
TNBC
triple-negative breast cancer

## Declarations

**Ethics approval and consent to participate:** This study was approved by The University of Texas MD Anderson Cancer Center Institutional Review Board (protocol LAB11-0337). All participants provided written informed consent for prospective research biopsy, molecular testing, and collection of clinical data in accordance with the Declaration of Helsinki.

**Consent for publication:** Not applicable.

**Data availability:** De-identified sequencing files are available upon request. Public deposition was not addressed in the original consent form, as it was not common practice at the time.

**Funding:** This work was supported by institutional research funds from The University of Texas MD Anderson Cancer Center (P30 CA016672); no external funding influenced study design, data collection, analysis, or interpretation.

**Authors' contributions:** B.L. conceived the study, conducted the data analysis, and drafted the manuscript. All authors reviewed, edited, and approved the final manuscript.

**Competing interests:** The authors declare no competing interests.

**Acknowledgements**

We thank the patients and families who participated in this study, as well as the Interventional Radiology and Molecular Diagnostics Laboratory teams at MD Anderson Cancer Center for their expertise in tissue acquisition and assay processing. We also acknowledge the support of the institutional Precision Oncology Decision Support team.

### Table 3

Table 3 is available in the Supplementary Files section.

### Figures

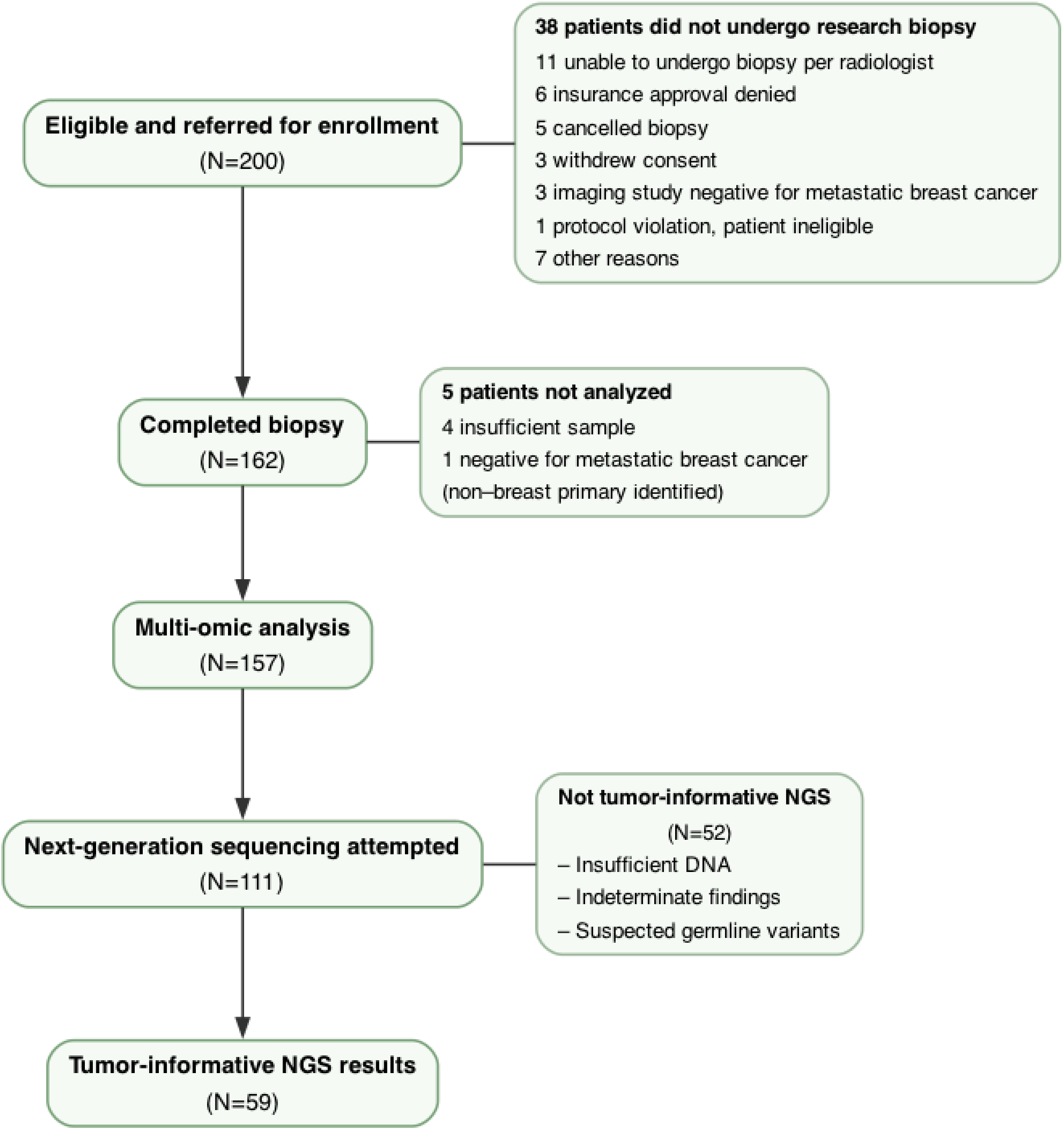

### Figure 1

#### CONSORT diagram

The CONSORT diagram depicts patient screening, biopsy completion, and analytic inclusion among individuals referred for enrollment between January of 2012 and October 2013. Of 200 eligible patients, 38 (19%) did not undergo a research biopsy due to radiologist-determined procedural infeasibility (n = 11), insurance authorization denial (n = 6), biopsy cancellation (n = 5), withdrawal of consent (n = 3), follow-up imaging negative for metastatic breast cancer (n = 3), protocol violation or ineligibility (n = 1), or other reasons (n = 7). Among the 162 patients who completed biopsy, 5 were excluded from downstream analyses because of insufficient tissue (n = 4) or identification of a non–breast cancer primary (non–small cell lung cancer; n = 1), resulting in a final analytic cohort of 157 patients who underwent multi-omic profiling. Next-generation sequencing was attempted in 111 samples, yielding tumor-informative results in 59 cases; 52 samples were not tumor-informative due to insufficient DNA, indeterminate findings, or suspected germline variants.

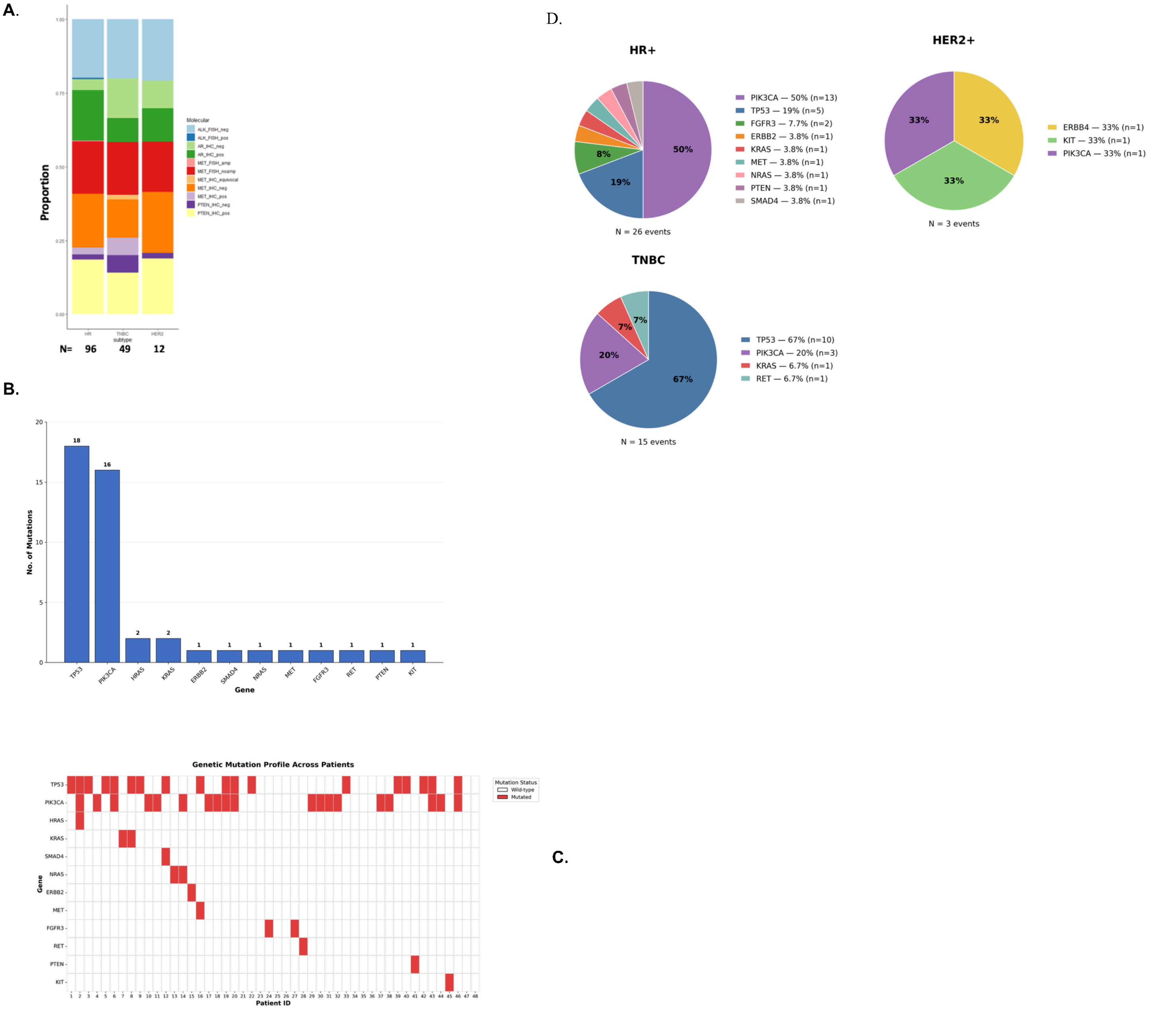

### Figure 2

**Integrative Genomic Landscape of the Study Cohort Stratified by Breast Cancer Subtype**.

A. Stacked bar chart showing the distribution of molecular markers across breast cancer subtypes: hormone receptor–positive (HR), triple-negative breast cancer (TNBC), and HER2-positive (HER2). Each bar represents the proportion of samples exhibiting specific molecular features, including ALK_FISH (negative/positive), AR_IHC (negative/positive), MET_FISH (non-amplified/amplified), MET_IHC (negative/equivocal/positive), and PTEN_IHC (negative/positive).

B. Frequency of recurrent somatic mutations identified in the study cohort. The bar chart displays the number of tumors harboring mutations in key genes, including TP53, PIK3CA, KRAS, FGFR3, ERBB2,

HRAS, KIT, MET, NRAS, PTEN, RET, and SMAD4. TP53 and PIK3CA were the most frequently mutated genes, with substantially lower mutation frequencies observed in other genes.

C. Heatmap illustrating the mutation status of selected genes across individual patients. Rows represent genes and columns correspond to individual tumors. Red squares indicate the presence of pathogenic or likely pathogenic mutations, highlighting inter-patient genomic heterogeneity within the cohort.

D. Pie charts depicting the distribution of genomic alteration categories across breast cancer subtypes (HR-positive, HER2-positive, and TNBC). Shown are the proportions of alterations classified as somatic mutations, variants of unknown significance, possible germline variants, and germline polymorphisms, illustrating subtype-specific differences in the genomic landscape.

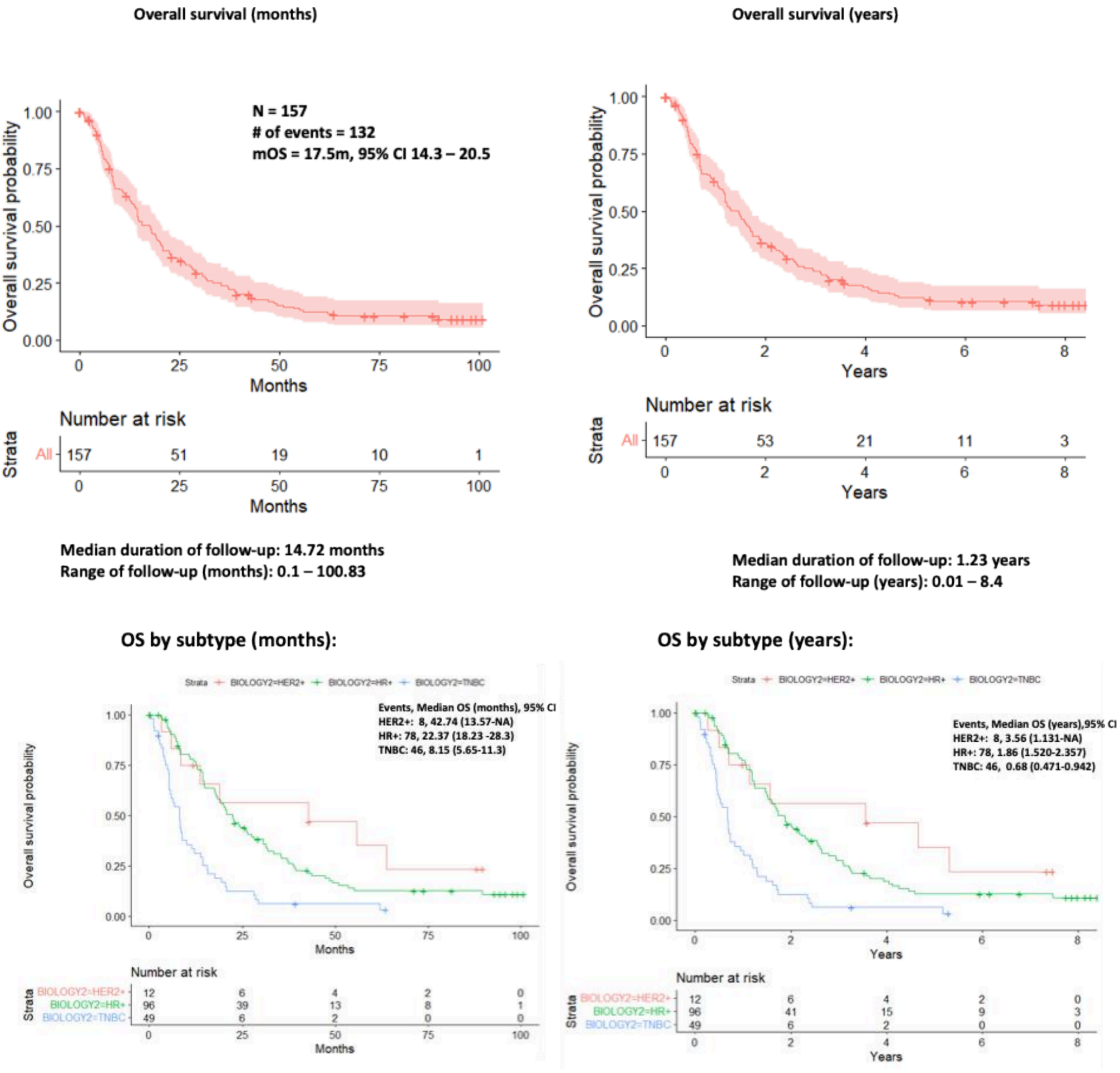

### Figure 3

#### Overall survival of study population and based on molecular subtypes

A.Kaplan–Meier estimates of overall survival for the full analytic cohort (N = 157). The left panel displays survival over time in months, and the right panel shows the same data in years for clinical interpretability. The solid red line represents the estimated survival function, with semi-transparent shading indicating the 95% confidence interval; red tick marks denote censored observations. Median overall survival (mOS) was 17.5 months (95% CI, 14.3–20.5), based on 132 death events. Numbers of patients at risk are shown below each time axis. Abbreviations: CI, confidence interval; mOS, median overall survival.

B.Kaplan–Meier overall survival stratified by breast cancer subtype: HER2-positive (red), hormone receptor–positive/HER2-negative (HR+, green), and triple-negative breast cancer (TNBC, blue). Tick marks indicate censored observations, and numbers of patients at risk are shown below each plot. At study entry, 12 patients had HER2-positive disease, 96 had HR+ disease, and 49 had TNBC. Median OS (95% CI) and number of events were: HER2-positive, 42.7 months (13.6–NA) / 3.56 years (1.31–NA), 8 events; HR+, 22.4 months (18.2–28.3) / 1.85 years (1.50–2.36), 78 events; and TNBC, 8.2 months (5.7–11.3) / 0.68 years (0.47–0.94), 46 events. Survival differed markedly by subtype, with progressively shorter survival observed in HR+ and TNBC compared with HER2-positive disease.

### Supplementary Files

This is a list of supplementary files associated with this preprint. Click to download.

- BEATITSupplementaryInformation.docx
- Table3.docx
